# The Potential of Selective COX-2 Inhibitor Mavacoxib for Extended Analgesia in Sheep Husbandry

**DOI:** 10.3390/ani16172791

**Published:** 2026-09-05

**Authors:** Oliver Fioretto, Lee Metcalf, Sabrina Lomax

**Affiliations:** 1 School of Life and Environmental Sciences, The University of Sydney, Camperdown, NSW 2006, Australia; ofio6534@uni.sydney.edu.au; 2 School of Veterinary Science, The University of Sydney, Camperdown, NSW 2006, Australia; lee.metcalf@sydney.edu.au

**Keywords:** nonsteroidal anti-inflammatory drug (NSAID), mavacoxib, animal welfare, husbandry procedures, livestock pain, sheep

## Abstract

Routine husbandry procedures in sheep induce significant and prolonged pain and inflammation, which often exceeds the duration of action of current commercially available analgesics. Mavacoxib is a selective cyclooxygenase-2 (COX-2) inhibitor approved for canine osteoarthritis treatment. With a half-life exceeding two weeks in dogs, mavacoxib may represent a candidate for investigating whether extended-duration analgesia can be achieved in sheep and more closely aligned with the duration of post-procedural inflammation and pain. This article examines the physiological and behavioural indicators of sheep pain, the limitations of current analgesic strategies, and the pharmacological rationale for mavacoxib as a candidate for pain management in sheep. Further research into its pharmacokinetics, efficacy, and safety in sheep is essential to improve welfare and overcome practical barriers to analgesia in extensive grazing systems.

## Simple Summary

Routine husbandry procedures like castration, tail docking and mulesing are essential for sheep management and health, but cause significant pain and inflammation lasting up to weeks. Despite this, pain management remains limited, with the most common medication only providing pain relief for up to 48 h. Mavacoxib is an anti-inflammatory drug currently used in dogs that provides long-lasting pain relief. Its extended action could better match the duration of pain in sheep compared to existing options. Research into mavacoxib for sheep could lead to improved animal welfare and more practical pain management on farms.

## 1. The Painful Reality of Routine Husbandry

With the ever-growing global demand for food and textiles, the sheep industry plays a crucial role in meeting agricultural needs. To improve animal health, productivity, and farm efficiency, routine husbandry procedures, such as ear tagging, castration, tail docking, and mulesing, are regularly performed. While these procedures serve important practical purposes necessary for health and productivity, they cause significant pain and inflammation due to surgical or ischaemic soft tissue damage [1,2], raising serious animal welfare concerns [3].

Despite a growing scientific consensus that these procedures cause considerable pain, they are often performed with inadequate pain management, if any at all. A survey by the Sheep Sustainability Framework [4] found that a substantial proportion of Australian farmers still do not use adequate analgesia during husbandry procedures. As societal expectations for animal welfare continue to evolve, husbandry procedures not only become ethically fraught without adequate analgesic and anti-inflammatory relief, but also biologically counterproductive. Unmanaged pain can suppress immune function, reduce feed intake, impair wound healing, and cause behavioural changes that disrupt flock cohesion and reduce productivity [2].

Currently, only six analgesic and anti-inflammatory drugs are registered for use in sheep in Australia [5]. Drug restrictions further limit accessibility to on-farm use by producers, with three of these needing to be administered by a veterinarian [5]. Additionally, the duration of post-procedural pain and inflammation often exceeds the duration of action of these drugs [6,7], with pain and inflammation lasting up to weeks [8]. This discrepancy between the biological duration of post-procedural pain and the pharmacological duration of currently available analgesics highlights one of the greatest limitations in sheep pain management. Investigating long-acting NSAIDs that better align with the inflammatory timeline should be a research priority so both animal welfare and on-farm productivity are significantly improved.

## 2. Inflammation and Its Consequences

### 2.1. Mechanisms of Inflammation

Acute inflammation is the body’s immediate response to tissue injury or infection, characterised by redness, heat, swelling, pain, and a loss of function at a tissue level [9]. Although associated with discomfort, inflammation is a protective process that promotes tissue repair by recruiting immune mediators such as cytokines, proteins and chemokines, which attract neutrophils and macrophages to the affected site [9]. The formation and release of pro-inflammatory mediators increase blood flow and fluid accumulation at the site, causing swelling and redness, and sensitise nerve endings, resulting in pain perception [10]. Pain acts as a protective mechanism by discouraging movement and further injury [11], thereby aiding in healing and recovery [12,13]. Because inflammatory mediators directly drive pain perception, quantifying physiological and behavioural consequences of inflammation provides objective methods for assessing pain severity and evaluating the efficacy of analgesic interventions [14,15].

**Figure 1:**
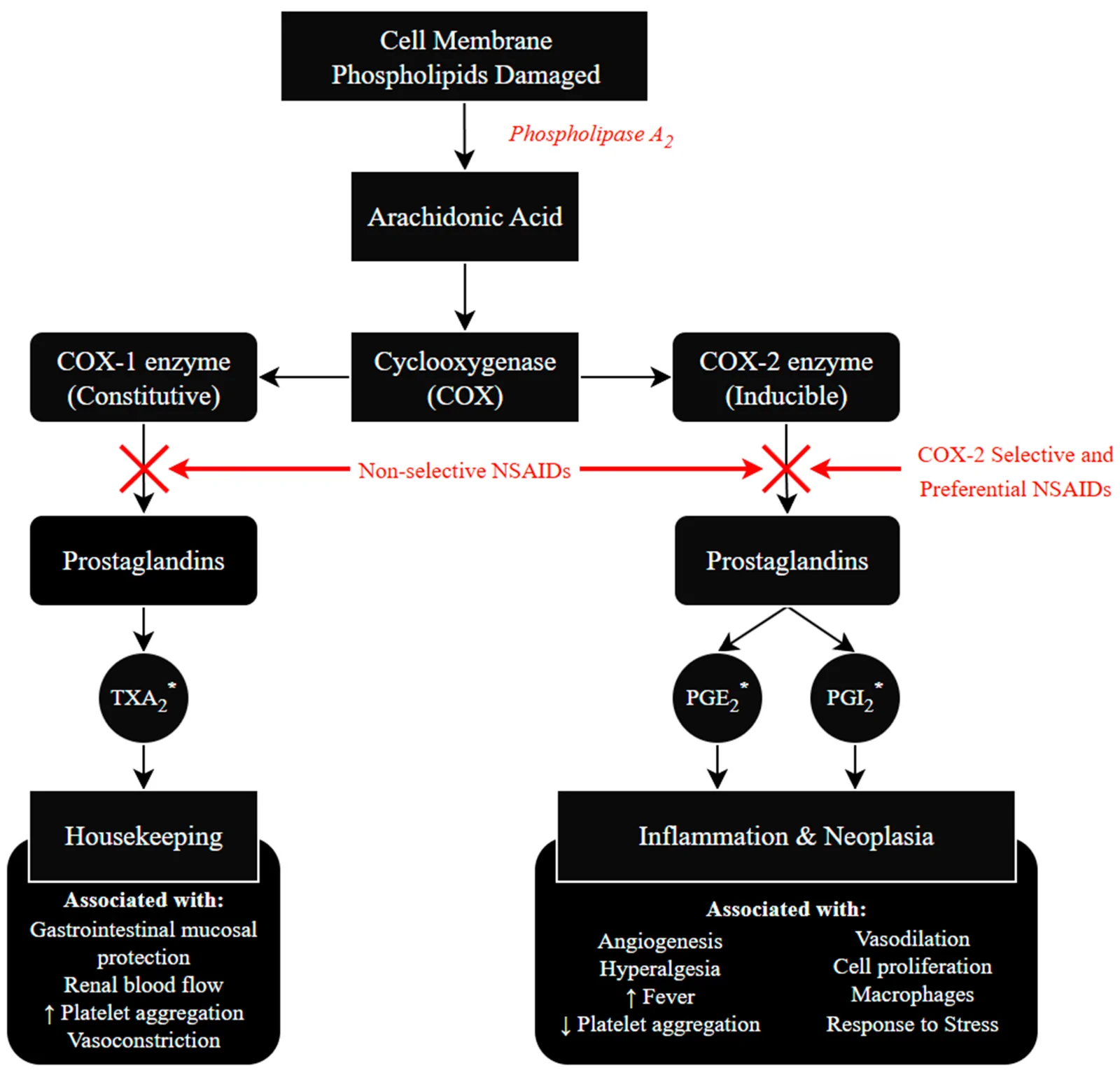
The arachidonic cascade into cyclooxygenase (COX) enzymes, COX-1 and COX-2, and their production of prostaglandins and the respective roles of their derivatives. TXA_2_ = Thromboxane A_2_; PGE_2_ = Prostaglandin E_2_; PGI_2_ = Prostaglandin I_2_; * = prostanoid more significantly produced by the respective COX enzyme it falls under. Red text indicates the inhibitory action of non-selective vs selective NSAIDs. Figure created by the authors based on information from Flood & Stewart [20], and Anwar et al. [21].

Among these inflammatory mediators, prostaglandins are of notable importance for their crucial role in regulating various bodily functions, including but not limited to inflammation and pain [16]. They are synthesised from arachidonic acid via cyclooxygenase (COX) enzymes (Figure 1), and contribute to nociceptor activation and peripheral sensitisation [14]. Of the two main COX isoforms, COX-2 is particularly relevant to husbandry procedures, as it is induced during injury and is a key driver of inflammatory pain [17]. Consequently, selective inhibition of COX-2 represents a logical pharmacological strategy for reducing inflammatory pain while minimising disruptions of normal physiological functions, making COX-2-selective NSAIDs attractive candidates for extended analgesia in sheep [17,18,19].

### 2.2. Signs of Inflammatory Pain in Sheep

Pain-related behaviours following husbandry procedures are well documented in sheep [2]. However, accurate assessment is challenging because sheep, as prey animals, instinctively mask signs of weakness or distress [15]. Pain expression in sheep is highly variable and influenced by individual and contextual factors, meaning identical painful stimuli can elicit different responses across animals. Age and sex affect pain sensitivity and behavioural expression, with older lambs displaying higher frequencies of some pain-related behaviours than younger lambs, and male and female lambs diverging in sensitivity during development [22]; notably, female lambs may exhibit more acute pain behaviours than males following tail docking [23]. Behavioural responses are further shaped by personality, prior experience, and social context; for example, the presence of familiar or related conspecifics can attenuate the expression of pain behaviours through social buffering, complicating interpretation in production settings [24].

Pain in sheep can be assessed using both physiological and behavioural indicators. Physiological markers such as elevated plasma cortisol, increased heart rate, and altered respiratory rate are commonly observed following painful interventions [25], but these responses lack specificity as they are also influenced by handling stress, environmental conditions, and social factors [15,25]. While often acute, physiological responses may remain elevated for extended periods following procedures such as mulesing and castration, indicating prolonged pain states [25]. Behavioural indicators include abnormal postures (e.g., hunched stance or lowered head), reduced activity, altered gait, bruxism, diminished grazing, increased vocalisation, and social withdrawal [15]. Facial expression-based measures, such as pain-specific grimace scales, provide an additional tool for detecting subtle changes in pain-related demeanour, although their application is currently more feasible in controlled environments than in field conditions [2].

Collectively, this demonstrates that pain in sheep is complex, variable and context-dependent, reinforcing the need for controlled approaches and validated experimental models to reliably and systematically study pain mechanisms and analgesic efficacy under reproducible conditions.

### 2.3. Experimental Models of Inflammatory Pain

Because behavioural and physiological responses to pain vary considerably between individual sheep, induced inflammatory models have been developed to provide a reproducible method for evaluating analgesic efficacy [26]. One established approach involves subcutaneous injection of oil of turpentine into the forelimb pastern, producing a localised inflammatory response that can be quantified using objective measures including limb circumference, skin temperature, pressure algometry, gait scoring, and weight-bearing assessment [26,27,28,29]. These models have successfully demonstrated the efficacy of meloxicam in reducing inflammatory pain [27,28] and provide a standardised platform for comparing novel analgesics.

However, the model primarily reflects acute inflammation and does not fully replicate the complex and prolonged pain experienced following routine husbandry procedures. Surgical tissue damage, ongoing wound healing, neuroimmune interactions, and environmental stressors [30,31] all contribute to post-procedural pain in commercial production systems and are not fully represented under controlled experimental conditions [32]. Resultingly, while experimental inflammation models provide an essential first step for evaluating candidate long-acting NSAIDs such as mavacoxib, complementary field studies following routine husbandry procedures remain necessary to determine whether prolonged pharmacological activity translates into clinically meaningful improvements in sheep welfare.

## 3. Current Analgesic Strategies and Their Limitations

### 3.1. Current Analgesic Options

Currently, only a limited number of products with analgesic effects are registered in Australia for pain mitigation associated with routine husbandry procedures in sheep [5]. These include injectable lignocaine as a local anaesthetic; Tri-Solfen^®^ (Dechra Veterinary Products Australia, Somersby, New South Wales), as a topical wound treatment (contains lignocaine, bupivacaine, adrenaline, and cetrimide); ketamine and xylazine as sedatives and anaesthetics; and meloxicam, available in both oral transmucosal and injectable forms and currently the only NSAID registered in Australia for use in sheep [5]. Although these products improve animal welfare by reducing procedural pain, they provide only short-term analgesia and therefore fail to adequately address the prolonged inflammatory response associated with many husbandry procedures [6,7,8].

This limited therapeutic toolkit poses a significant challenge for effective pain relief in extensive sheep production systems. Inadequate analgesia not only compromises animal welfare but also has broader implications, including prolonged cortisol elevation, which can suppress immune function and reduce feed intake and weight gain [33]. These effects ultimately reduce productivity and increase economic losses, further highlighting why the importance of accessible and effective pain mitigation strategies in livestock remains a priority [2].

### 3.2. The State of Current Analgesia

One of the greatest limitations of current analgesic strategies is the mismatch between the biological duration of post-procedural pain and the pharmacological duration of available drugs. Pain and inflammation following routine husbandry procedures such as castration, tail docking and mulesing can persist for several days to weeks, with behavioural and physiological changes frequently persisting to the end of observation periods in published studies [8]. In contrast, currently available analgesics provide substantially shorter periods of pain relief. Although meloxicam is currently the only NSAID registered for use in sheep in Australia [5] and measurable plasma concentrations persist for up to 48 h following administration [28], the duration of clinically effective analgesia is less well established and is unlikely to match the prolonged inflammatory period associated with these procedures, as currently understood from behavioural and physiological studies [6,7,8,34].

Maintaining therapeutic analgesic concentrations throughout the period of inflammation would therefore require repeated administration. While this approach is common in companion animal practice, where dogs routinely receive NSAIDs for several days following surgery [35], repeated dosing is often impractical in extensive sheep production because animals must be re-mustered and rehandled, increasing labour requirements, handling stress, and concerns regarding residue management [36,37]. These practical constraints likely contribute to the low uptake of pain relief by producers, with surveys reporting that 75% of producers do not use analgesia for castration and 56% do not use it for tail docking [4]. However, uptake is likely multifactorial and may also be influenced by product cost, ease of administration, residue withholding-period concerns, and producers’ perceptions of the necessity or benefit of analgesia, rather than duration of action alone [36,37].

Collectively, these limitations demonstrate that current analgesic strategies are constrained not only by limited drug availability but also by insufficient duration of action. This mismatch highlights the need for practical, single-dose NSAIDs capable of providing prolonged analgesia that more closely aligns with the biological course of inflammation. Long-acting COX-2 inhibitors therefore represent a logical next step in improving pain management for sheep undergoing routine husbandry procedures.

## 4. Mavacoxib: A Promising Long-Acting Analgesic?

### 4.1. Pharmacological Rationale and Safety Considerations for Use in Sheep

Mavacoxib is a highly selective COX-2 inhibitor from the COXIB class of NSAIDs, and is currently approved only for the treatment of canine osteoarthritis [38]. It has also been investigated in rabbits [39] and some avian species [40,41,42,43]. Although its pharmacokinetics and safety have not yet been established in sheep, its pharmacological profile makes it an attractive candidate for livestock analgesia.

Cyclooxygenase enzymes regulate prostaglandin synthesis from arachidonic acid. COX-1 primarily maintains physiological homeostasis, whereas COX-2 is predominantly upregulated following tissue injury and drives inflammatory pain [17,18]. Consequently, selective COX-2 inhibition reduces inflammation while largely preserving normal COX-1-mediated physiological functions, making COXIBs particularly attractive for prolonged pain management [44].

In-vitro assays using canine blood demonstrate that mavacoxib is between 37.8- and 128-fold more selective for COX-2 than COX-1 [19,45], compared to meloxicam, which is only 12-fold more selective [46]. These selectivity ratios were generated in canine in-vitro assays and should not be assumed to apply directly to sheep, because COX-1/COX-2 selectivity estimates are assay- and species-dependent; equivalent sheep-specific selectivity data for mavacoxib are not currently available. This profile is desirable for pain management in livestock, as it targets the inflammatory pathways directly while minimising off-target effects associated with COX-1 inhibition, such as gastrointestinal ulceration and renal dysfunction [21,47,48].

While efficacy is critical, NSAID use also depends on safety, as prostaglandin inhibition underlies both therapeutic and adverse effects. Adverse reactions most commonly affect the gastrointestinal tract, kidneys, and liver [49], with gastrointestinal lesions presenting as irritation, erosion, or ulceration, potentially causing anaemia, protein-losing enteropathy, or melaena [50,51]. In dogs, mavacoxib’s (Trocoxil 20 mg chewable tablets for dogs) most frequently reported adverse reactions are vomiting and diarrhoea, with uncommon reports of appetite loss, haemorrhagic diarrhoea and melaena, and rarely observed gastrointestinal ulceration [52]. Although COX-2-selective NSAIDs can induce ulceration in monogastric species [47,52], there is no evidence of NSAID-induced abomasitis or intestinal perforation in ruminants [6,53]; notably, one study reported that cattle given 30 times (30 mg/kg) the recommended dose (1 mg/kg) of meloxicam showed no adverse effects or gross histologic lesions [51]. While the long half-life of mavacoxib raises considerations regarding tissue persistence and the potential for extended withholding periods in food-producing animals [36], these factors may be less restrictive if its use were targeted to young animals following routine husbandry procedures, which are typically performed many months before slaughter. Nevertheless, determination of residue depletion profiles and establishment of appropriate maximum residue limits would remain essential prerequisites for any registration pathway. Despite these regulatory considerations, its favourable pharmacodynamic profile suggests strong potential as a candidate for safe, effective, welfare-aligned analgesia in sheep, pending further investigation of its pharmacokinetics, residue profile and commercial feasibility.

Beyond efficacy and safety, the potential application of mavacoxib in sheep would require consideration of regulatory and residue-related challenges associated with food-producing animals. The prolonged persistence that makes mavacoxib attractive as an analgesic may also result in extended tissue residues and lengthy withholding periods. Therefore, residue depletion studies, maximum residue limit determination, and food safety assessments would be required before commercial adoption could be considered. If mavacoxib were developed for use in sheep, practical on-farm considerations would also include the appropriate dose, route and frequency of administration, whether treatment could be readily performed by producers or would require veterinary involvement, and the feasibility of a simple single-dose intervention. The duration of any withholding period would depend on sheep-specific pharmacokinetic, residue-depletion, formulation and regulatory data and therefore cannot currently be established. These practical considerations should be addressed in future experimental and translational studies alongside efficacy and safety.

### 4.2. The Pharmacokinetics of Mavacoxib

Pharmacokinetic data from dogs show that mavacoxib demonstrates a remarkably low clearance rate of 0.0027 L/hr/kg, ~130-fold lower than the low clearance rate threshold for dogs of the same weight (0.348 L/hr/kg) [54], and a prolonged half-life of 17.3 days [38]. Its lipophilicity further suggests extensive distribution into inflamed tissues, offering targeted analgesia at wound sites [18], a favourable characteristic for procedures like castration and tail docking. The low clearance rate and minimal renal excretion, with elimination predominantly via biliary secretion and faecal excretion [18], distinguish mavacoxib from NSAIDs such as meloxicam and carprofen, which rely on renal and faecal pathways [34,55,56]. This also means accumulation may occur in animals with hepatic impairment, explaining its contraindication in dogs with liver disease [57]. Its long half-life is further supported by high plasma protein binding, large volume of distribution, and enterohepatic recycling [58]. However, the exceptionally low clearance and prolonged half-life observed in dogs should not be assumed to occur in sheep. Clearance and terminal half-life can differ noticeably between species, particularly between monogastric and ruminant species, because gastrointestinal and ruminal physiology, plasma protein binding, hepatic metabolic capacity, bile flow, and enterohepatic cycling may substantially alter drug disposition [59,60]. Although hepatic metabolism and biliary excretion may also contribute to mavacoxib elimination in sheep, their quantitative importance cannot be assumed to be conserved from dogs. Only empirical pharmacokinetic studies in sheep can determine mavacoxib clearance, systemic exposure, terminal half-life, and the duration for which therapeutically relevant concentrations are maintained. Consequently, direct pharmacokinetic extrapolation from dogs to sheep is not appropriate without empirical evaluation. Nevertheless, the known pharmacological characteristics of mavacoxib support investigation of whether prolonged systemic exposure may be achievable in sheep and whether this could translate into clinically meaningful analgesia.

### 4.3. Addressing the Inflammation Duration Mismatch in Current Analgesics

If mavacoxib demonstrates prolonged systemic exposure and pharmacological activity in sheep, it could offer significant welfare advantages by more closely aligning analgesia with the extended inflammatory response associated with husbandry practices, which often span several days to weeks [6]. This contrasts with meloxicam, which possesses a much shorter half-life (10.82 ± 2.46 h in sheep after subcutaneous dose of 1 mg/kg; [28]) and a significantly higher clearance rate (0.008 ± 0.002 L/hr/kg in sheep after subcutaneous dose of 1 mg/kg; [28]) [34] and requires frequent redosing to sustain therapeutic efficacy [34]. However, the canine-ovine clearance comparison is hypothesis-generating only; it cannot establish the magnitude or duration of mavacoxib exposure in sheep. Improved alignment between pharmacokinetics and inflammatory windows may promote greater compliance with pain relief protocols, improve animal welfare, and potentially enhance growth performance and productivity outcomes [2]. Whether such alignment can actually be achieved with mavacoxib must be established through sheep-specific pharmacokinetic and pharmacodynamic studies before conclusions about duration of action can be drawn. Collectively, these features highlight the strong potential of mavacoxib for integration into livestock pain management strategies, warranting further investigation.

## 5. Conclusions

Routine husbandry procedures in sheep continue to present a significant animal welfare challenge because the duration of post-procedural pain frequently exceeds the duration of action of currently available analgesics. Although existing NSAIDs improve welfare, their relatively short duration of activity limits their ability to provide sustained pain relief under extensive production systems, where repeated administration is often impractical.

Mavacoxib represents a promising candidate to address this pharmacological mismatch. Its prolonged half-life and high COX-2 selectivity demonstrated in dogs provide a rationale for investigation, but these properties cannot be assumed to translate quantitatively to sheep. Its favourable pharmacokinetic profile suggests that it may provide extended analgesia more closely aligned with the biological duration of inflammation than currently available options. However, its efficacy, safety, pharmacokinetics, residue profile, and optimal dosing regimen remain unknown in sheep.

Future research should prioritise pharmacokinetic, pharmacodynamic, residue-depletion, and safety studies in sheep, followed by controlled efficacy trials and commercial field validation. Determining whether mavacoxib can safely provide prolonged analgesia following routine husbandry procedures will be essential before any clinical or commercial application can be considered.

Although significant knowledge gaps remain, the mismatch between the duration of post-procedural pain and the duration of action of currently available analgesics highlights an important unmet need in sheep welfare. Mavacoxib represents a biologically plausible candidate for addressing this gap and warrants further investigation as part of ongoing efforts to improve pain management in extensive livestock production systems.

## Data Availability

No new data were created or analysed in this commentary; therefore, data sharing is not applicable.

## Abbreviations

The following abbreviations are used in this manuscript:

NSAID	Nonsteroidal anti-inflammatory drug
COX	Cyclooxygenase
